# Enhanced drug delivery and wound healing potential of berberine-loaded chitosan–alginate nanocomposite gel: characterization and *in vivo* assessment

**DOI:** 10.3389/fpubh.2023.1238961

**Published:** 2023-12-27

**Authors:** Md Habban Akhter, Lamya Ahmad Al-Keridis, Mohd Saeed, Habibullah Khalilullah, Safia Obaidur Rab, Adel M. Aljadaan, Mohammad Akhlaquer Rahman, Mariusz Jaremko, Abdul-Hamid Emwas, Sarfaraz Ahmad, Nawazish Alam, Md Sajid Ali, Gyas Khan, Obaid Afzal

**Affiliations:** ^1^School of Pharmaceutical and Population Health Informatics (SoPPHI), DIT University, Dehradun, India; ^2^Department of Biology, Faculty of Science, Princess Nourah Bint Abdulrahman University, Riyadh, Saudi Arabia; ^3^Department of Biology, College of Science, University of Hail, Hail, Saudi Arabia; ^4^Department of Pharmaceutical Chemistry and Pharmacognosy, Unaizah College of Pharmacy Qassim University, Unaizah, Saudi Arabia; ^5^Department of Clinical Laboratory Sciences, College of Applied Medical Sciences, King Khalid University, Abha, Saudi Arabia; ^6^Department of Pharmacology, College of Pharmacy, Najran University, Najran, Saudi Arabia; ^7^University of Nottingham Graduate Entry Medicine, Royal Derby Hospital, Nottingham, United Kingdom; ^8^Department of Pharmaceutics and Industrial Pharmacy, College of Pharmacy, Taif University, Taif, Saudi Arabia; ^9^Division of Biological and Environmental Sciences and Engineering (BESE), King Abdullah University of Science and Technology (KAUST), Thuwal, Saudi Arabia; ^10^Core Labs, King Abdullah University of Science and Technology (KAUST), Thuwal, Saudi Arabia; ^11^Department of Clinical Pharmacy Practice, College of Pharmacy, Jazan University, Jazan, Saudi Arabia; ^12^Department of Pharmaceutics, College of Pharmacy, Jazan University, Jazan, Saudi Arabia; ^13^Department of Pharmacology and Toxicology, College of Pharmacy, Jazan University, Jazan, Saudi Arabia; ^14^Department of Pharmaceutical Chemistry, College of Pharmacy, Prince Sattam Bin Abdulaziz University, Al-Kharj, Saudi Arabia

**Keywords:** wound healing, polymer, nanomedicine, nanotechnology, chitosan, alginate, nanocomposite, berberine

## Abstract

Berberine–encapsulated polyelectrolyte nanocomposite (BR–PolyET–NC) gel was developed as a long-acting improved wound healing therapy. BR–PolyET–NC was developed using an ionic gelation/complexation method and thereafter loaded into Carbopol gel. Formulation was optimized using Design-Expert® software implementing a three-level, three-factor Box Behnken design (BBD). The concentrations of polymers, namely, chitosan and alginate, and calcium chloride were investigated based on particle size and %EE. Moreover, formulation characterized *in vitro* for biopharmaceutical performances and their wound healing potency was evaluated *in vivo* in adult BALB/c mice. The particle distribution analysis showed a nanocomposite size of 71 ± 3.5 nm, polydispersity index (PDI) of 0.45, ζ–potential of +22 mV, BR entrapment of 91 ± 1.6%, and loading efficiency of 12.5 ± 0.91%. Percentage drug release was recorded as 89.50 ± 6.9% with pH 6.8, thereby simulating the wound microenvironment. The *in vitro* investigation of the nanocomposite gel revealed uniform consistency, well spreadability, and extrudability, which are ideal for topical wound use. The analytical estimation executed using FT-IR, DSC, and X-ray diffraction (XRD) indicated successful formulation with no drug excipients and without the amorphous state. The colony count of microbes was greatly reduced in the BR–PolyET–NC treated group on the 15th day from up to 6 CFU compared to 20 CFU observed in the BR gel treated group. The numbers of monocytes and lymphocytes counts were significantly reduced following healing progression, which reached to a peak level and vanished on the 15th day. The observed experimental characterization and *in vivo* study indicated the effectiveness of the developed BR–PolyET–NC gel toward wound closure and healing process, and it was found that >99% of the wound closed by 15th day, stimulated via various anti-inflammatory and angiogenic factors.

## 1 Introduction

Approximately 4.5 million individuals in the US alone undergo medical care for chronic wound and an estimated cost of nearly USD$ 25 billion is spent every year in the management of chronic wound therapy. However, the impediment due to chronic wounds is growing day by day with every increase in the incidence of diabetes and obesity (1).

Skin is the largest organ of the body, and it acts as a protective barrier for internal organs against threats of environmental hazards. The excise area or injuries over the skin surface can be healed after it goes through several physiological and biological upshots. Physicochemical features of the wound environment, such as, pH and temperature, may vacillate with the level of inflammation, the level of microbial infection, and aeration to the wound area (2). The normal temperature of wound area varies from 32°C to 34°C or higher depending on the level of inflammation. Post-excising of the skin, temporary pH was reported to be 7, and this pH may decrease to acidic levels of 4 throughout the stages of healing. The decrease in pH also depends on the level of microbial infection (3). Wound healing generally restores the damaged tissues through a plethora of changes involving, intially, the phase of hemostasis that occurs post-injury, followed by the phases of inflammation, proliferation, and remodeling of tissues. Following hemostasis, fibrin triggers clot formation in the wound matrix and platelets start releasing several growth factors to initiate the healing process. The inflammation phase starts within 24 h and may last upto 2 weeks in normal injury or longer in case of chronic wound. Macrophages, neutrophils, and monocytes are key cells in the inflammation phase. These cells act as phagocytes to clean the cell debris and further release pro-inflammatory mediators that involve fibroblast formation and re-epithelization of the wounds (4). Subsequent to this stage, a proliferative phase involving the synthesis of fibroblast cells, collagens, and the extracellular matrix and, finally, a remodeling phase triggering the basic alteration in collagen organization, replacement of scar tissues with an organized normal extracellular matrix occur. Besides these two phases, other factors affect wound healing such as oxygen supply to the growing tissue, age, degree of infection, cytokines, nutrition, hormones, production of ECM, and enzyme proteases (5, 6). Due to the dynamic characteristics of the wound environment, the controlled drug release via an active targeted drug delivery system is an efficient treatment option to be explored over the traditional hypodermic injection approach, which enable an increase in drug concentration outside the above therapeutic indications and tends to cause side effects and reduce efficacy (7, 8). Furthermore, passively targeted nanocarriers enable the increase in drug concentration for a longer duration, thereby maintaining drug concentration in the blood for the therapeutic window to obtain efficient recovery of the wound surface (9–11).

Berberine (BR) is an isoquinoline alkaloid extract obtained from the rhizomes of plants such as *Phellodendron amurense*, *Berberis aristata*, and species of *Coptis* (12). A considerable increase in interest in exploring the medicinal alkaloid has been observed worldwide due to their potential biological activity. Previous literature reported that berberine may exhibit anti-inflammatory (13), anti-oxidant (14), anticancer (15), anti-microbial (16), anti-diabetic (17), anti-hypertension (18), antiviral (19) properties and may be used for the treatment of mood disorders (20). The naturally existing hydrophobic feature and limited aqueous solubility raise questions about BR’s effectiveness (20). However, several attempts have been made in the past to enhance their dissolution and oral bioavailability, such as polysaccharide-based nanoparticles for enhanced oral bioavailability (21), berberine liposomes for oral delivery (22) and cardiac therapy (23), chitosan-layered nanoliposome for oral administration (24), and pharmacokinetic evaluation and hypoglycemic effect of berberine loaded with solid lipid nanoparticles (25). The anti-inflammatory and anti-oxidative properties of benzylisoquinoline alkaloide berberine (BR) is regulated by many molecular pathways, including the inhibition of the mitogen-activated protein kinase (MAPK) signaling pathways (26). Berberine has shown anti-inflammatory properties both *in vitro* and *in vivo*. It suppresses the gene transcriptions associated with interleukin-1, interleukin-6, and tumor necrosis factor-α and reduces the concentration of inflammatory proteins. Berberine also inhibits cyclooxygenases, thereby inhibiting prostaglandin production (27).

The alginic acid forms a complex with chitosan via electrostatic attraction of the chitosan molecules via its γ-carboxyl moiety. The chitosan–alginate complex protects encapsulated drug molecules from biodegradation in the biological system (28). The chitosan–alginate complex enhanced the rate of BR dissolution and the permeability of BR. The process of ionic polymerization accelerated when brought in contact with calcium ions through binding with alginate guluronic residues, resulting in the formation of polyanionic nanoparticles (29). Several studies reported the use of chitosan as a natural polymer in wound healing application (30–33). Nanoparticles have been used in nano therapy as a carrier for the delivery of a large number of therapeutic regimens for several diseases over the past decade, and their substantial use in the modern therapeutic system owing to overwhelming responses based on tissue selectivity and specificity, improving drug concentration to the target, and minimizing off-target delivery is notable (34).

## 2 Materials and methods

### 2.1 Materials

Berberine (BR) was purchased from Sisco Research Laboratories, which also (Mumbai, India) provided sodium alginate and chitosan (MW–50–190 KDa, 85% deacetylation). Various analytical grade reagents such as HPLC water, solvents, and other chemicals were used in the analysis. The components of the solution used for buffer preparation/phosphate saline buffer (PBS) were of analytical grades. The Central Drug House Pvt. Ltd. (CDH, New Delhi) provided sodium dihydrogen phosphate, potassium dihydrogen phosphate, sodium hydroxide, disodium hydrogen phosphate, glacial acetic acid, dichloromethane, acetonitrile, and ethanol.

### 2.2 Optimization using Design-Expert

Using the BBD in Design-Expert® software (Version 10; Stat-Ease Inc., Minneapolis, Minnesota), we generated 17 formulations for experiments implementing three levels and three factors for optimizing the formulations (35–37). The levels of independent factors were decided considering the outcomes of the preliminary test in the laboratory, which was classified into minimum level (−1), intermediate level (0), and higher level (+1), as summarized in the Table 1. Chitosan (X1), sodium alginate (X2), and calcium chloride (X3) as independent factors were 40 mg, 60 mg, and 80 mg; 20 mg, 30 mg, and 40 mg; and 20 mg, 30 mg, and 60 mg, respectively corresponding to the levels (−1), (0), and (+1). Using the BBD in Design-Expert® software, 17 formulations were generated, which underwent an extensive characterization process for the responses PS (y1) and entrapment efficiency (y2). The details of the obtained response analysis of individual factors are shown in Table 2. The details of the individual and interaction effects of the input attributes on the response were sorted out. This design was often helpful in sorting the problem of selecting three or more independent variables as the analysis requires an elite group of experimental runs than what is required for the central composite design (38). The primary aim of using the Design-Expert software was to optimize the formulation and make it stable and robust by enchancing improved entrapment efficiency, drug loading, and higher drug release concomitantly with reduced particle size and PDI. The ANOVA test was performed to validate polynomial equations generated by the Design-Expert software, which was validated using the ANOVA test. All collected responses were provided from the use of the Design-Expert software. Various possibilities in the experimental run were explored to obtain the optimum composition of the formulation BR-PolyET-NC. The Design-Expert software generated 3D surface morphology, contour plots, and the predicted-to-observe response of selected dependent variables to demonstrate the significant impact of excipients under use on dependent variables of the formulation.

**Table 1:** The formulation variables and the levels employed as low (0), medium (−1), and high (+1) in optimizing BR–PolyET–NC.

Independent variables	Level employed		
	Low (−1)	Medium (0)	High (+1)
X1: Chitosan (mg)	40	60	80
X2: Sodium alginate (mg)	20	30	40
X3: Calcium chloride (mg)	30	45	60
Dependent variables			
Y1: Particle size (nm)	Maximize		
Y2: Entrapment efficiency	Maximize		

**Table 2:** The different experimental run (1–17) predicted by in the BBD for responses Y1 and Y2 for the optimization of BR–PolyET–NC.

	Factor 1 = X1	Factor 2 = X2	Factor 3 = X3	Response = Y 1	Response = Y2
Run	A:Chitosan	B:Sodium alginate	C:Calcium chloride	Particle size	Entrapment efficiency
	mg	mg	Mg	nm	%
1	80	30	60	120	60
2	60	20	30	70	69
3	40	20	45	90	67
4	40	30	60	85	60
5	60	20	60	60	68
6	80	30	30	120	60
7	60	30	45	65	88
8	80	40	45	140	60
9	80	20	45	90	69
10	60	40	28	90	60
11	40	40	45	80	65
12	60	30	45	62	87
13	40	30	30	90	60
14	60	30	45	59	89
15	60	40	60	85	60
16	60	30	45	70	87
17	60	30	45	62	89

A, chitosan (mg); B, sodium alginate (mg); X3, calcium chloride (mg); Y1, particle size (nm); Y2, drug encapsulation.

### 2.3 Preparation of BR-loaded-nanocomposite

A nanocomposite of BR-loaded alginate–chitosan nanoparticles (BR–PolyET–NC) was prepared using the ionic gelation technique modifying the preceding study (39). A solution of sodium alginate (10 mL) was first prepared in deionized water (30 mg/mL) and a pH of 5.2 ± 0.02 was maintained. The BR (10 mg) vortex was dissolved in 96% ethanol and then incorporated dropwise into the alginate solution, ensuing the preparation of a homogeneous pregel solution. Then, 5 mL of an aqueous calcium chloride solution was supplied dropwise to the alginate solution and stirred continuously using a magnetic plate at 1,000 rpm for 30 min, leading to the formation of the alginate complex gel. Chitosan (50 mg/mL) was separately thawed in 1% v/v acetic acid; then, 10 mL of this solution was imparted in the alginate solution dropwise and stirred using a magnetic plate thereafter, following which the plate was sonicated for 10 min. Then, the chitosan-coated NPs were harvested after centrifugation at 15,000 rpm for 40 min and finally obtained the alginate–chitosan complex (Alg–Ch complex), which was lyophilized for characterization (39).

### 2.4 Nanocomposite characterization

#### 2.4.1 Particle size, polydispersity index, and surface charge

The particle size of synthesized BR–PolyET–NC was measured using Malvern Zetasizer instrument (Nano ZSP, Malvern Instruments, Worcestershire, United Kingdom). The working of the instrument was based on the dynamic light scattering (DLS) technique. When a beam of light passed through the particle of the samples, it scattered due to the Brownian movement or the zigzag motion of sample particles. The scattering phenomenon was observed at a scattering angle of 90° at room temperature. The NPs were extracted from the gel formulation by diluting in Milli-Q grade water at a ratio of 1:100, vortexed, and sonicated to reach homogeneous consistency. The particle size was measured in triplicate (*n* = 3). The surface charge on the BR–Ach–NC was also measured.

#### 2.4.2 Percentage BR entrapment and loading

The BR-loaded nanocomposite, BR–PolyET–NC, was centrifuged at 12,000 rpm for 20 min. The aliquot of suspension was pulled out and analyzed at λmax of 422 nm in the UV spectrophotometer for free BR concentration. The amount of BR entrapped and BR loading in the nanocomposite was determined using equations given below.


(3) $$\%EE=\frac{\left(\text{Total}\;BR\;\text{added}-\text{Total}\;BR\;\text{in}\;\text{aliquot}\right)}{\text{Total}\;BR\;\text{added}}\;\times 100$$

(4) $$\%DL=\frac{\left(\text{Total}\;BR\;\text{added}-\text{Total}\;BR\;\text{in}\;\text{aliquot}\right)}{\text{Total}\;\text{weight}\;\text{of}\;\text{nanocomposites}}\;\times 100$$

#### 2.4.3 Nanocomposite FT-IR and x-ray diffractometer

A total of 5–10 mg of the BR–PolyET–NC sample was used for FTIR spectral analysis (BRUKER Corporation, Billerica, MA, United States). The sample was kept in contact with a beam of light that emerged from the FTIR instrument, and the sample was scanned over the wave number range of 4,000–500 cm^−1^. XRD was performed for BR, alginate, chitosan, and physical mixture and optimized gel applying diffractometer (PANalytical X’pert PRO, Netherland). The measures of electric current from the x-ray tube of the XRD were 40 kV and 100 mA during the process of Cu Kb reduction using a nickel-filter. The sample scanned at 2θ angle ranged from 10 to 50°, at a speed limit of 10°/min.

#### 2.4.4 Transmission electron microscopy

Transmission electron microscope further evaluated the particle size with a TEM instrument (Techni TEM 200 Kv, Fei, Electron optics). For electron microsope examination, a drop of the diluted sample was disseminated onto the copper grid (carbon-coated) and the grid was coated with phosphotungstic acid. Finally, the developed sample was examined under the microscope.

#### 2.4.5 Percentage drug release and kinetic study

BR release from the BR–PolyET–NC nanocmoposite was carried out in simulated intestinal fluid (SIF) along with PBS with pH of 6.8 and then compared with BR dispersion. Then, the study nanocomposite was passed through the activated dialysis membrane in PBS. The measured quantity of BR–PolyET–NC was 10 mg, and the BR gel was enwrapped in a dialysis bag with a PBS volume of 95 mL (MW; 8–12 kDa; Repligen, Waltham, MA, United States) with its ends closed and transferred into dissolution medium. The dissolution medium was maintained at a physiological temperature of 37 ± 0.5°C with uninterrupted stirring (40, 41). The requisite sample volume of 1 mL in various time frames, that is, 0, 8, 16, 24, 32, 40, and 48 h, were withdrawn, and the exact volume of fresh buffer was put back in dissolution medium. The collected samples were suitably treated (i.e., filtered and diluted) and quantified using UV-spectrophotometry. BR release from the carrier was interpreted through plotting data between %drug release versus time. Furthermore, the incurred drug release data fitted into various mathematical models, and a good model fit for BR release from the nanocomposite was predicted. The mathematical equations expressing different models are shown below.


(5) Mt/Mo=K×t→Zeroorder

(6) $$\text{or},\;\ln\left(\frac{M}{Mo}\right)=K\times t\to \text{Zero}\;\text{order}$$

(7) $$\frac{Mt}{Mo}=K\times t\frac{1}{2}\to \text{Higuchi}\;\text{model}$$

(8) $$\frac{\;Mt}{Mo}=K\times tn\to \text{Korsemeyer}-\text{Peppas}$$

where, K represents a kinetic rate constant, Mt./Mo indicates the fraction of drug released at time t, and n is the diffusion exponent. The *n*-value expresses the mechanism of drug release, herein, *n* ≤ 0.5 (Fickian diffusion), 0.5 < *n* < 1.0 (Anomalous, non-Fickian transport), and *n* = 1.0 (relaxation Case-II).

#### 2.4.6 Fabrication of nanocomposite gel

The BR-loaded nanocomposite, i.e., BR–PolyET–NC gel, was developed by compounding and mixing using a propeller-type mixer 1% BR–PolyET–NC in previously dissolved 1% Carbopol 940 in distilled water, which is followed by the addition of 1% propylene glycol and preservative, 0.01% methyl paraben, and, thereafter, few drops of triethanolamine to the mixture. The mixing process was continued until a clear, transparent gel has not been formed, and its pH was adjusted to make it compatible with the physiological skin pH. The development of the chitosan–alginate nanocomposite gel using polymer Carbopol 940 grades was carried out in accordance with our previously reported study with some modifications (36).

#### 2.4.7 Gel characterization

##### 2.4.7.1 Organoleptic features, drug content, viscosity, and pH

The BR–PolyET–NC gel was examined visually for colour, odor, and taste. A digital meter was employed for measuring the pH of the gel (Thermo Scientific, Waltham, MA, United States). Gel pH was measured in triplicate (*n* = 3). The drug content can be expressed as the percentage of drug showcased in gel preparation. It is the ratio of the drug in the nanocomposite to the true value of the drug loaded on the gel. The viscosity profile of the developed gel was determined by applying the shear rate (1/s).

##### 2.4.7.2 Gel homogeneity, spreadability and extrudability

The homogeneity of the developed gel was investigated via visual inspection by placing the gel in a clear glass beaker kept in a fixed position. The spreadability of the gel was tested by sandwiching 0.5 g of the gel in between two glass plates, and the gel spread to a diameter of 1 cm. A weight box of 0.5 kg was then set above the plate for 5 min, and, thereafter, spreading of the gel under the influence of weight was ascertained. The spreading area of the gel was determined using a Vernier caliper (*n* = 3). The limit of the gel’s spreadability was considered to be 2 cm^2^ for topical application (42). An aluminum collapsible tube was completely filled with 15 g of the gel from the bottom with care to prevent the entrapment of air. After filling, the bottom side was folded in triplicate and then compressed under a crimping machine, and then a plastic cap was wrapped on the top side of the tube and sealed. The process of extrusion was commenced after removing the sealed cap from the tube followed by a gentle pressing of the tube. Gel extrudability was determined using following formula.


(9) $$\text{Extrudability}=\frac{\text{Weight}\;\text{applied}\;to\;\text{the}\;\text{tube}}{\text{Area}\;\left(\text{cm}2\right)}$$

##### 2.4.7.3 Surface morphology

Nanocomposite gel was spread on an aluminum stub, air-dried, then gold coated, and finally observed under a JSM 6100-Digital Scanning Electron Microscope (JEOL, Ltd. Tokyo, Japan).

### 2.5 *In vivo* studies

For this study, BALB/c rats weighing between 200 and 230 g were procured. The studies were performed in ethical compliance to the research guidelines approved by the Institutional Animal Ethics Committee (IAEC) of DIT University (Dehradun, Uttrakhand, India under ref. no. DITU/IAEC/22/04/28) dated 26 September 2022. The animals were placed inside polypropylene cages that were kept under 12-h light/dark cycles at ambient temperature and relative humidity was maintained. Animals were categorized into four groups (*n* = 4). Surgical incisions were made on the rat skin. On the day following surgical incision, treatment protocol was followed with the BR–PolyET–NC gel, and BR dispersion occurred topically twice a day until the 15th day, which may be compared either to untreated control or negative control. On completion of the treatment protocol, animals were euthanized, and sections of skin tissues were stored in 10% formalin at −20°C for histopathological investigation.

#### 2.5.1 Macroscopic wound area closure measurement

Wound contraction percentage (%woundcontraction) was determined after measuring the diameter with a scale and, thereafter, using the following formula:


(10) $$\%\text{Wound}\;\text{contraction}=\frac{\begin{array}{c} \text{Wound}\;\text{diameter}\;\text{on}\;5\text{th}\;\text{day}\\ -\text{Wound}\;\text{diameter}\;\text{on}\;15\text{th}\;\text{day} \end{array}}{\text{Wound}\;\text{diameter}\;\text{on}\;5\text{th}\;\text{day}}\times 100$$

#### 2.5.2 Microbial assessment

The swabs excluded from the wound part of the skin on every 5th, 10th, and 15th days of wound dressing change. Using the serial dilution technique for quantitative investigation of bacterial colon count, swabs were diluted by 10-fold using normal saline. A total of 500 μL of each diluted specimen was kept on 2% agar medium spread on petri plates and incubated at physiological temperature for 24 h. After incubation time, the colony count of bacterial generation was investigated.

#### 2.5.3 Preparation of wound tissue specimen

On 5th, 10th, and 15th days, mice from different groups were captured after removing the dead tissues from the wound site, and these tissues were taken for microscopic analysis to investigate the pattern of re-epithelization, fibrogenesis, and collagen formation.

#### 2.5.4 Histopathological analysis

For this analysis, after euthanizing the animals, the wounded area was carefully removed and rinsed with saline water, and the skin tissue was dissected and preserved in formalin (10%). Thereafter, sections of approximately 5 μm thickness were paraffinized using a microtome with prior treatment with xylene and ethanol. Then, the specimens were treated to remove a paraffin layer, hydrated, and stained using hematoxylin and eosin (43). The prepared slides were examined by a pathologist who was blindled to the origin of the specimens using a CX31 microscope (Olympus Corporation, Tokyo, Japan) connected with a NanoZoomer-SQ Digital slide scanner C13140-01 (Hamamatsu Photonics, Shizuoka, Japan).

### 2.6 Stability studies

The developed optimized gel preparation in a glass container well covered with aluminum foil was subjected to stability tests under refrigerated condition (5 ± 3°C) and at elevated temperature (40 ± 2°C/75 ± 5% RH) in stability chamber as per International Conference on Harmonization (ICH) guidelines. The formulation was inspected at regular intervals on days 0, 30, and 60 for specific physico-chemical changes such as particle size, PDI, pH, viscosity, particle size, PDI, spreadability, and extrudability. In addition, physical appearence features such as clarity, turbidity, and phase separation were also inspected visually.

### 2.7 Statistical analysis

The samples were analyzed using a one-way ANOVA plus the Tukey–Kramer test using GraphPad Prism (version 7). The data were represented in the form of mean ± SD (*n* = 3). The significance level was considered at a value of *p* of <0.05.

## 3 Results and discussion

### 3.1 Optimization

**Figure 1:**
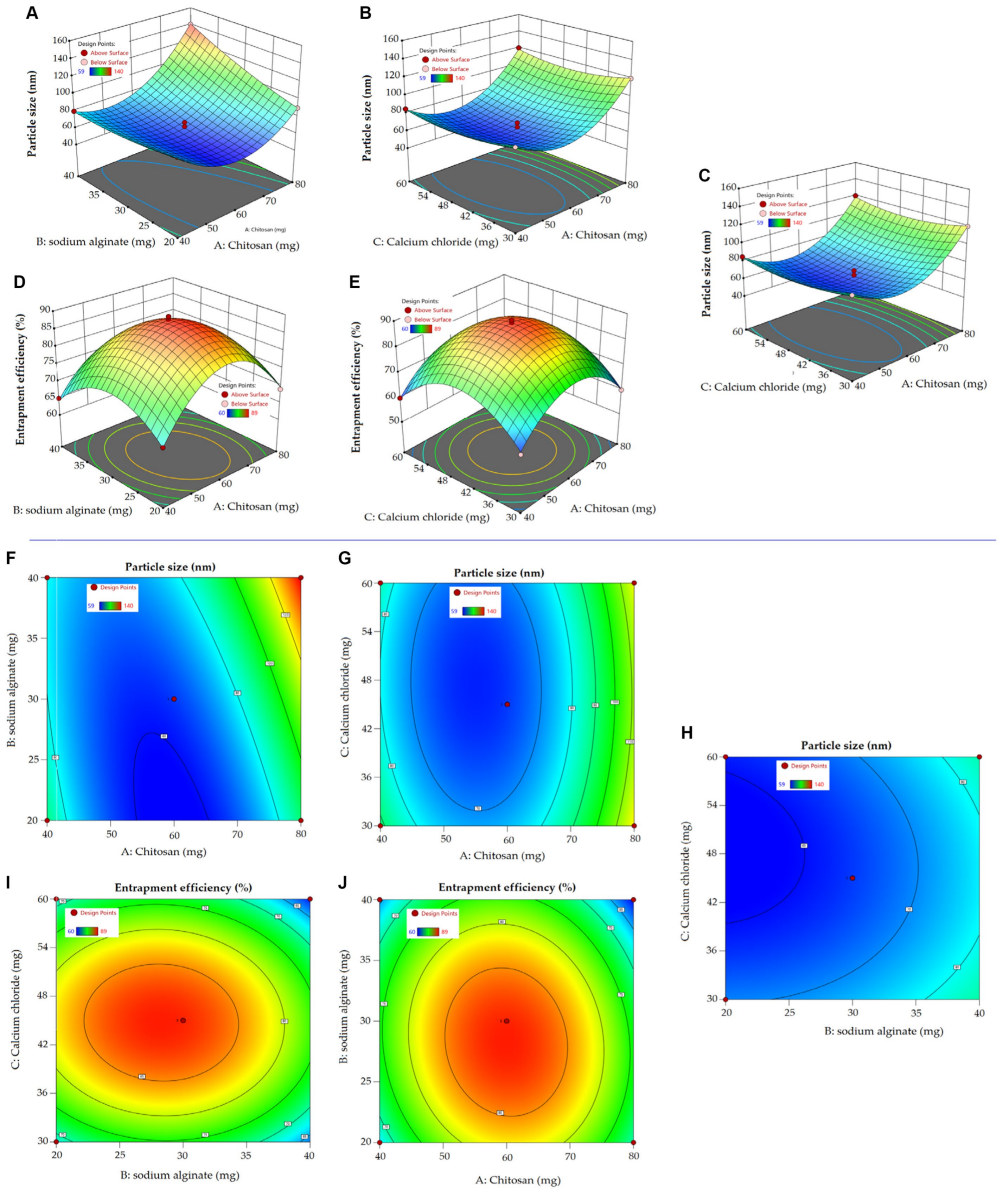
Response surface curve **(A–E)** exploring the impact of independent variables on dependent variables in the preparation of NPs. Two dimensional contour curve **(F–J)** exhibiting the influence independent variables have on dependent variables of NPs.

The present study aimed to develop, characterize, and evaluate the effectiveness of a BR-loaded chitosan–alginate polyanionic complex gel in treating self-induced wound in rat model. Employing a three-factor, three-level Box Behnken design (BBD), the formulation was optimized. The various levels of independent variables are shown in Table 1. Response surface morphology and contour plots demonstrating the influence of excipients on particle size and %EE are shown in Figures 1A−E and Figures 1F−J. Contour plots and response surface morphology demonstrating the influence of excipients on particle size and %EE are shown in Figure 1. The quadratic model was determined as the best-fitted model with a high coefficient of correlation (*R*^2^) of approximately 1 among the other 2FI and linear models while considering the significant influence of independent variables on responses. A polynomial equation established according to the best fit model guidelines well explained the different components on independent variables combined with the quadratic effect on dependent variables. Seventeen formulations were developed in the BBD, accommodating five center points to advertently check for any replicas (05) of these formulations (Table 2) (35).

### 3.2 Impact of X1, X2, and X3 on particle size (Y1) and % EE (Y2)

The optimum relationship between chitosan concentration (X1), sodium alginate (X2), and calcium chloride (X3) as independent variable and factors, namely, particle size and % EE, are displayed below.


(1) $$\begin{array}{l} \text{Particle}\;\text{size}=+63.57+15.63\times X1+10.33\times X2-2.23\times X3\\ \;+15.00\times X1\times X2+1.25\times X1\times X3+1.79\times \\ \;X2\times X3+32.24\times X12+4.15\times X22+7.99\times X32 \end{array}$$

(2) $$\begin{array}{l} \%EE=+87.99-0.375\times X1-3.75\times X2-0.606\times X3\\ \;-1.75\times X1\times X2-0.711\times X2\times X3-13.98\\ \;\times X12-8.78\times X22-14.00\times X32 \end{array}$$

The quadratic equation (1) shows a positive impact of chitosan and sodium alginate on particle size. The particle size ranges between 58 to 140 nm, indicating a uniform and narrow distribution. The smallest particle size obtained was 58 nm at a chitosan concentration of 60 mg and the highest particle size obtained was 140 nm at a chitosan concentration of 80 mg. Increasing chitosan concentration may increase the bulk of the polymer in the formulation, resulting in bigger particles (44). The high viscosity of the formulation may lead to binding to the alginate gel matrix that result in increased particle size (45). Furthermore, sodium alginate had a positive effect on the particle size due to the formation of a complex with positive charges bearing chitosan. In an aqueous sodium alginate solution, calcium ion replaces sodium ion to form calcium alginate, thereby forming a calcium alginate complex. Cross-linker Ca^2+^ ions provide elasticity and, thus, stabilize the nanocarrier (46). Ahdyani et al. pointed out that raising Ca^2+^ ion concentration in chitosan-alginate formulation led to their reduction in chitosan-alginate NPs (39). In addition to the individual effect, the combined effect of chitosan and alginate was positive on particle size. 3D response surface curve and 2D contour plots well explicated the impact of independent variables on response particle size (Figure 1).

In terms of particle size, %EE is an important parameter under consideration in the development of a successful nanoplatform. The impact of an independent variable on %EE is comprehensively explained in equation (2), with 3D response surface curve and contour plot. The encapsulation of BR in various developed formulation ranges from 60 to 89%. The formulation with the highest chitosan concentration showed a slightly reduced entrapment of BR. A high chitosan concentration might increase the viscosity of the preparation solution, causing the barrier to deliver the BR inside the matrix core of the polymer. Increasing the concentration of sodium alginate in the preparation solution led to a slightly reduced % EE. Zimet et al. prepared Nisaplin®-entrapped alginate NPs through the ionic gelation complexation technique. They observed reduced %EE aside from an increase in particle size from 86 to 204 nm and a zeta potential from −33.2 to −38.7 mV (47). The cross-linker Ca^2+^ ion marginally reduced the %EE of the chitosan–alginate NPs, probably due to being in contact with porous hydrophilic sodium alginate in the aqueous medium (48).

### 3.3 Checkpoint analysis

The numerical optimization technique revealed the optimized composition of BR formulation, restraining minimum particle size (Y1) and maximum % EE. The desirability value close to 1, i.e., 0.987, elaborates the stable and coherent nature of the formulation. The smaller the particle size, the large will be the surface area, thereby contributing to better solubility and dissolution of the drug and, hence, improving overall drug availability from the nanosystem in the biological medium (36). The optimized formulation, BR–PolyET–NC, comprised of a chitosan concentration of 58.8 mg, a sodium alginate concentration of 27 mg, and a calcium chloride concentration of 45.27 mg. The model anticipated value for the responses particle size and %EE were noted as 65 nm and 88%, respectively. The experimental estimated value of the responses particle size and % EE were 71 ± 3.5 nm and 91 ± 1.6%, respectively. Less variation in the model anticipated value determine by software and experimental value was observed, which was not statistically significantly different (*p* > 0.05), as expressed in Table 3. Furthermore, the surface charge on the surface of the NPs was estimated to be +22 mV, indicating a stable optimized formulation. Moreover, the low polydispersity index (PI) shows a homogeneous and uniform and narrow distribution of nanosized particles throughout the developed formulation (40).

**Table 3:** Independent variables, estimated value, and model predicted value of optimized the BR–PolyET–NC gel.

Independent variables	Response variables	Optimized formula	Estimated value of responses	Predicted response	Anticipated error†
X1: Chitosan (mg)	Particle size (nm)	58.5 mg	71 ± 3.5 nm	65 nm	8.4
X2: Sodium alginate (mg)	EE (%)	27.0 mg	91 ± 1.6%	88%	3.29
X3: Calcium chloride (mg)	-	45.27 mg	-	-	-

†Calculated as (estimated value – predicted value/predicted value)/estimated value × 100.

### 3.4 Characterization of optimized formulation

#### 3.4.1 Nanoparticle characterization, BR entrapment, and BR loading

**Figure 2:**
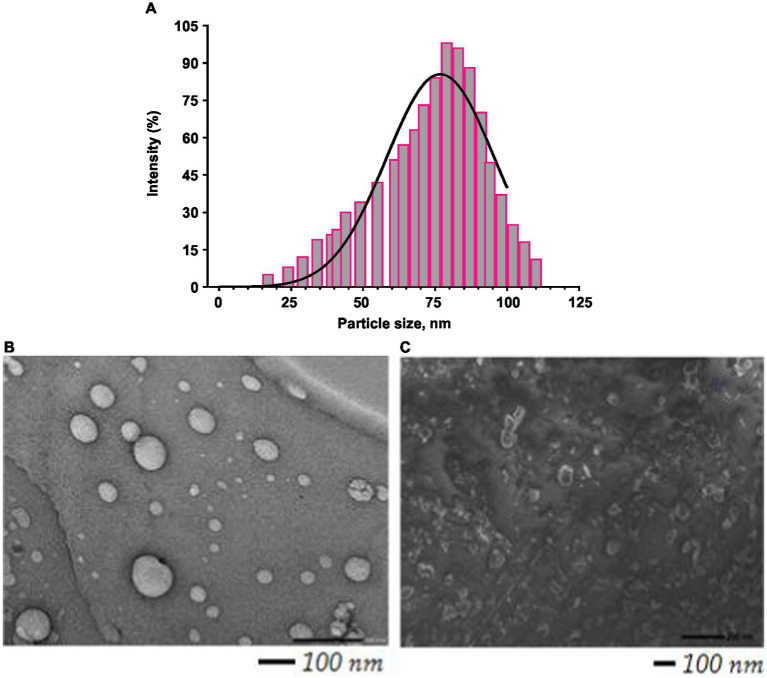
Histogram showing particle size distribution curve **(A)**; TEM image of BR–PolyET–NC **(B)**; SEM image of BR–PolyET–NC gel **(C)**.

The particle size of BR–PolyET–NC observed to be 71 ± 3.5 nm, and the particle population in the formulation was uniform and consistent and unimodal. The PI value of <0.5 could be considered as the monodispered system. The polydispersity index of formulation was low, that is, 0.45, which suggested homogeneity and monodispered nanosize system (Figure 2A). The surface charge on BR–PolyET–NC was reported to be +22 mV using Malvern Zetasizer Nano ZSP. The positive surface charge on the chitosan NPs was due to the presence of the cationic functional group, and it provides the colloidal stability to the NPs. The surface charges help to integrate the cell membrane *in vivo* (49). The high percentage of BR entrapment (91 ± 1.6%) and loading (12.5 ± 0.91%) was estimated.

#### 3.4.2 Percentage drug release and kinetic model

**Figure 3:**
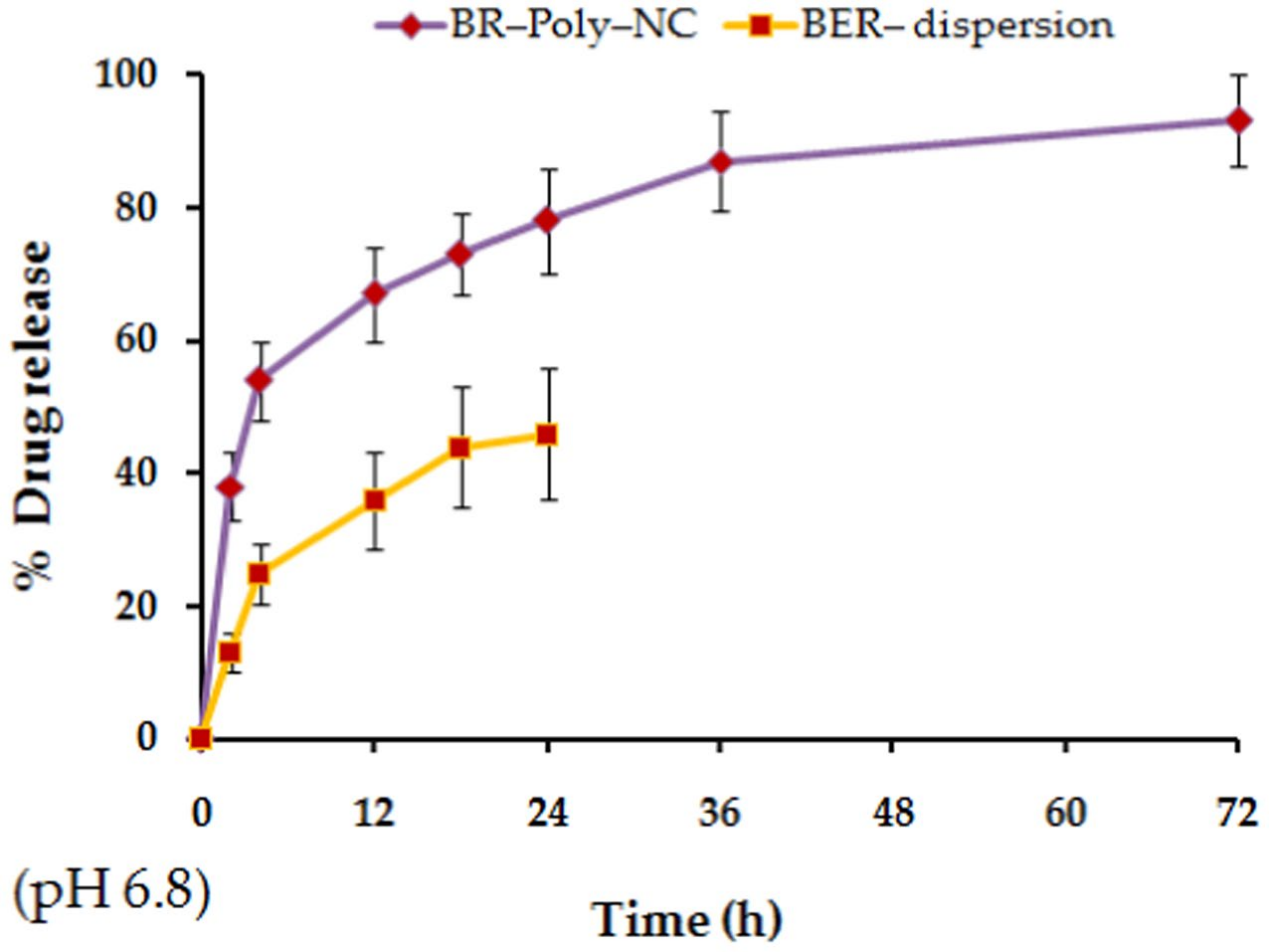
Percentage of BR release from BR–PolyET–NC and BR-dispersion at pH 6.8.

Percentage release of BR from BR–PolyET–NC and BR-dispersion at predefined time points has been investigated, as shown in Figure 3. The drug release characteristics follows the biphasic pattern, where it appears that an abrupt release of the drug ahead of time abide by the sustained release model from the nanocomposite mentioned at the end of the study. In the initial 2 h of injection, a fast drug release was noticed, followed by a slow and sustained release in a controlled way for a period of 72 h. The burst release was due to adsorbed or weakly held drug particle on to theNP surface, and BR-entrapment and dispersion were estimated to be 38 ± 5.19% and 13 ± 3% in the initial 2 h at room temperature in pH 6.8. The poor release of BR from BR dispersion may be ascribed to the hydrophobicity of the drug, resulting in low aqueous solubility and dissolution. The maximum BR release from the nanocomposite after the completion of 72 h was observed to be 89.50 ± 6.9% at pH 6.8 compared to 46 ± 10% the BR release from BR dispersion. Overall, % cumulative release from BR–PolyET–NC was apparently high over drug dispersion. A higher release from the nanocomposite may be attributed to the straightaway dissolution and then drug diffusion at pH 6.8. The dissolution study reported herein is in concurrence with previous studies in the literature (50, 51).

The polysaccharide nanocomposite of alginate–chitosan, bearing Contrary surface charge or opposite surface charge, has been widely explored in drug delivery and biomedical application (52). Owing to the swelling property of polysaccharides, chitosan is more repulsive in an environment of positive charge, which thereby initiates the movement of positive ions from chitosan and negative ions from alginate in their combined complexes with calcium, with Ca^2+^ ion triggering improved drug release. The PolyNC formed primarily due to the ionic interaction of chitosan with alginate in the presence of divalent cations encapsulate protection of the surface by reducing pores in the complex and thus enhancing the controlled release of BR. The prompt solubility of chitosan at slightly acidic pH and contrarily high solubility of alginate at neutral pH in the nanocomposite concomitantly led to the sustained release of BR, which is an ideal feature for improved therapy at the wound site. Despite the good sustainability of BR through the gel system, the bioadhesive nature of chitosan prolonged the abidance of BR at the wound site, minimized rapid clearance of the drug, and thereby improved the efficacy of BR at the target (44, 53). The alginate fraction of the complexes showed sustained release and improved longer-acting BR release. Furthermore, BR release from the alginate–chitosan nanocomposite complex was enhanced by aqueous penetration into the polyelectrolyte complex, causing gel swelling, matrix erosion, and, therefore, drug diffusion into the circumferential medium. Additionally, drug release from the nanocomposite also relies on pH of the dissolution medium, physicochemical properties of the drug and nanocarriers, and nanocarrier interaction with the biological system at the wound surface (41).

The BR release of the nanocomposite was fitted to kinetic models of first order, and Higuchi, Korsmeyer–Peppas, and Hixson–Crowell were screened out as the best models of good fit. The coefficient of correlation (R2) of the selected kinetic model was estimated, and it indicatded that the model of good fit for BR release from the nanocomposite was Korsmeyer–Peppas, with highest value of R2 being 0.9632. Furthermore, the n exponent value was determined to be 0.398 (0.5 < n < 1), revealing Fickian diffusion of BR from the polymeric composite, BR–PolyET–NC (52).

#### 3.4.3 Nanocomposite XRD and FTIR spectral analysis

**Figure 4:**
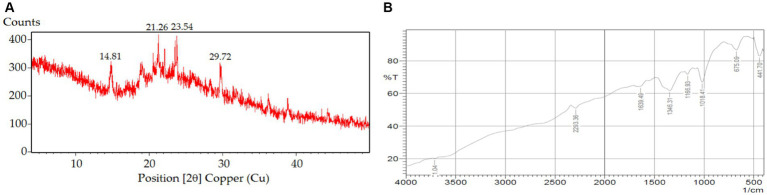
**(A)** XRD, and **(B)** FTIR of berberine nanocomposite.

XRD is an important analytical parameter to illustrate the molecular state of drug encapsulated in the nanoparticles. The physical state of the drug indicates the extent of solubility, dissolution, absorption and thus reflects the bioavailability of the drug from the formulation. The XRD peaks of BR-loaded nanocomposite clarified that some of the berberine peaks could be noticed in XRD of the nanocomposite (Figure 4A). These peaks were observed in reduced intensity at 2Θ of 14.81, 21.26°, 23.54° and 29.72°. Based on the diffraction pattern of the compound, BR has been greatly reduced in the nanocomposite, stating the amorphization or conversion into molecular BR in the formulation (37, 54, 55). All the characteristics peaks of berberine appeared in the formulation, although their intensity slashed to zero level or reduced, which affirmed the encapsulation of the alcohol and carboxylate groups or other groups of the compound berberine in the chitosan–alginate nanocomposite. Although some reduced characteristic peaks appeared at 2,293.36 cm^−1^ due to the C=C bond stretching, at 1,639.49 cm^−1^ due to C=O stretching, at 1,346.31 cm^−1^ due to C–C stretching, and at 1,166.93 cm^−1^ due to N-H stretching. The peak shown at 1,037.70 cm^−1^ is due to C–O stretching (alcoholic and carboxylic) (Figure 4B). Thus, the FTIR spectrum of nanocomposite substantiated the drug excipients’ stability in the formulation.

#### 3.4.4 Measurements of particle size distribution and electron microscopy

Particle size and their distribution in the bulk of nanosize preparation are shown in Figure 2A. The TEM image shows that particle size is uniform, and the spherical, scattered, and agreeable size distribution was uniform and monodisperse with prior investigation with Malvern Zetasizer. A TEM study revealed the particle distribution was 71 ± 3.5 nm (Figure 2B). NP in this range is assorted with antimicrobial activity at the wound site. The SEM image revealed a spherical morphology of uniform consistency and was non-porous (Figure 2C).

#### 3.4.5 Characterization of BR–PolyET–NC gel

##### 3.4.5.1 Organoleptic features, drug content and pH

The color of the gel appeared pale yellow, and the odor was acceptable. Drug content in the gel was reported to be 99 ± 0.50%. The high drug content in the gel indicated the reliable method of high accuracy in gel preparation and that is desirable for pharmaceutical gel. A digital pH meter measured the pH of the developed gel to be 6.3. The ideal pH range of topical gel preparation was 4.5–6.5. A higher pH value of the gel may set off scaly skin, and low pH may set off dermatitis. The deviation in pH of the gel may cause modification in the skin pH preceding the adverse impact on the cutaneous barrier layer, dermal microflora, and also the normal healing mechanism of skin (56). A nanocomposite gel comprised of the gelling agent is Carbopol 940. The current preparation used a Carbopol (an acrylic polymer) concentration of 1%, which produced a transparent, consistent, and stable preparation and showed better permeation or drug release. Polyethylene glycol was used as a humectant and solubilizer. Methylparaben was used as a preservative to prevent microbial growth. Trithanolamine neutralized the caboxylic group from carbomer and maintained the desired pH and it also acts as an emulsifier and thickening agent.

##### 3.4.5.2 Viscosity of BR–PolyET–NC gel

**Figure 5:**
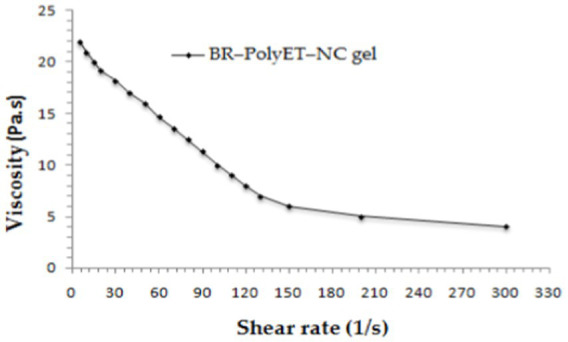
Viscosity (Pa.s) versus shear rate (1/s) profile of BR-PolyET-NC gel.

BR–PolyET–NC gel homogeneity was inspected visually by placing it in a settled position in a container. It was of uniform consistency and homogeneous with no aggregates. The viscosity of the BR–PolyET–NC gel was determined to be 9.23 Pa.s. Applying the shear rate (1/s) indicating that the shear-thinning system means the application of shear force, which led to decreased viscosity of the gel system, which helped in easy application to the target site (Figure 5).

##### 3.4.5.3 Gel homogeneity, spreadability and extrudability

The developed gel was visually observed to be homogeneous in consistency when settled in a container. It was reported to be free of any particle aggregates. The spreadability test was performed to estimate the nature of flow of the gel, and it indicated good flowability in the range of 5–7 cm. Gel spreadability was determined to be 6.2 ± 0.12 cm, indicating better dissemination and suitability in topical application. Good spreadability of the gel after application at the wound or disease site ensures easy spread over an area and cover of the whole wound in less time (~2 s) and thus helps in effective and fast healing. Furthermore, the extrudability of the gel was measured to be >90%, indicating excellent extrudability. Gel retention was excellent without draining at the wound site after application and thus ensures better adherence (57).

### 3.5 *In vivo* wound healing activity

#### 3.5.1 Macroscopic wound area closure measurement

**Figure 6:**
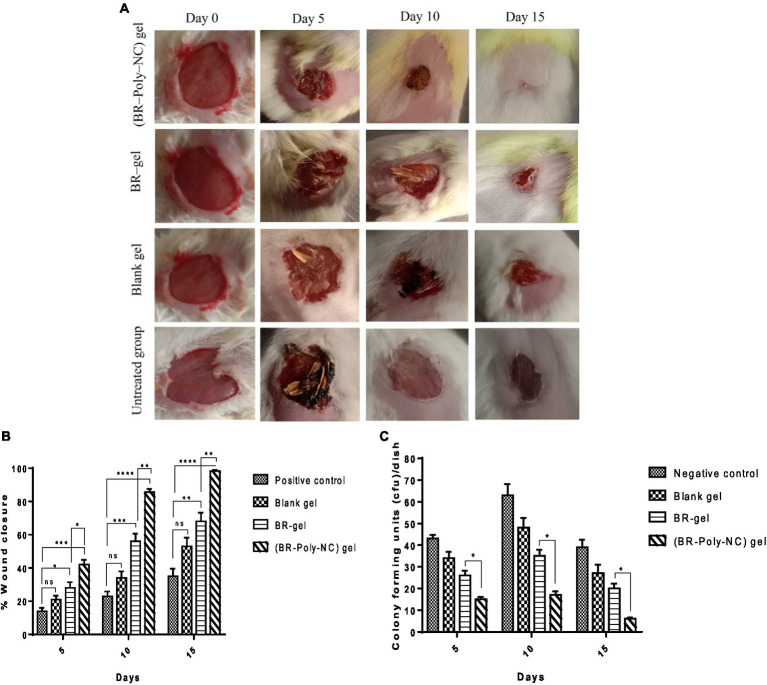
**(A)** Time dependent (0, 5, 10, and 15 days) healing of the wound in Balb C mice following treatment with BR–PolyET–NC, BR gel, blank gel, and positive control (untreated). **(B)** Wound closure measured on days 5, 10, and 15 after incision made on skin following treatment with BR–NP gel, BR gel, and blank and positive control. **(C)** Microbial colony count at the wound site. Data were expressed as mean ± SD (mean ± SD = 3). Observations among the group were statistically compared using a one-way ANOVA and Tukey’s multiple comparison test. The level of significance; *p < 0.05, **p < 0.01, ***p < 0.001, ****p < 0.0001 when compared to different groups.

The macroscopic features of various treated groups of animals with BR–PolyET–NC gel, BR gel, blank gel, and untreated/positive control for the wound healing progress and closure are shown in Figure 6A. As shown in the Figure 6B, among the entire treated group of the wound, the BR–PolyET–NC gel treated group had their wound closed at approximately the 15th day. On 5th, 10th, and 15th days, the wound closure percentage was reported to be 42 ± 5%; 85.55 ± 3.5%; and 98.22 ± 1%, respectively, in the animals in the BR–PolyET–NC gel treated group. Similarly, in the BR gel and blank gel treated groups, wound closure percentage was determined to be 68 ± 9% and 55 ± 9% on the final day of treatment. In the case of the control group (untreated), only 35 ± 8% wound closure was measured on the 15th day. The BR–PolyET–NC gel reduced the wound gap significantly compared to the BR, blank, and control gels on every 5th, 10th and 15th days (Figure 6B).

It is believed that berberine exert the anti-inflammatory action in wound healing process, which primarily relies on the signalling pathway of NF-кB, that is, silent information regulator 1 (Sirt1). The wound healing mechanism of berberine involves upregulation of the NF-κB protein Sirt1 and thus lead to decreased expression of NF-κB, inhibiting TNF-a and IL-6 expression and decreasing inflammatory protein expression. The reduced inflammatory response altered the wound microenvironement by increasing the expression of VEGF and CD31 and eventually wound healing progressed. Sirt1 also directly involves reversal of the inflammatory cells and anti-oxidant stress (58). Berberine as a wound healing agent has been recommended by various researchers. A few illustrative examples have been indicated in this study. Panda et al. recently developed BR–loaded lecithin–chitosan nanosystem for wound healing in diabetic rats. The findings of these authors suggested that combining chitosan and berberine together in a carrier gave synergistic effect toward wound healing. These authors further observed that optimum formulation worked by eliminating inflammatory cells and increasing mature collagen fibers (44).

Yin et al. developed a berberine-decorated zinc oxide colloid nanohydrogel (ZnO-Ber/H) for the wound healing activity in diabetic rats. The developed gel had an excellent wound closure rate of 92.9% by the end of the 15th day. The developed preparation ZnO-Ber/H downregulated inflammatory factors, upregulated vascular factors, and improved the re-epithelization in the wound (59).

Zhang et al. developed BR nanohydrogel of alginate. The molecular mechanism of healing relied on BR-mediated activation of Sirt1, which, in turn, promotes wound healing by reducing inflammatory factors and progressing angiogenesis (58). In addition, Samadian et al. developed a berberine-loaded electrospun cellulose acetate/gelatin mat in treating diabetic foot ulcers (60). The nanofiber produced a mean diameter of 502 ± 150 nm. *In vivo* studies disclosed that CA/Gel bandage dressing exhibited their antibacterial potential and improved collagen density by 8.8 ± 6.7% and received an angiogenesis score of 19.8 ± 3.8. Thus, an electrospun mat bearing BR proved to be helpful in wound healing activity in animals. Moreover, Amato et al. developed a nanogel consisted of BR, hyaluronan, and poly-L-lysine (61). The results expressed that a nanogel encapsulating BR reduced the fibroblast gap after 42 h. Zhou et al. proved that TrxR1 plays an important role in governing redox homeostasis in different pathologic conditions. The BBR works on TrxR1 was elucidated. BBR outstandingly promotes the synthesis of the extracellular matrix and remarkably damages HaCaT cells in the wound healing process. Furthermore, BBR activated TrxR1, leading to the suppression of downstream JNK signal and preventing oxidative stress and apoptosis, and thus raised cell proliferation, growth factor, amd tissue inhibitors and later lowered matrix metalloproteinase, thus promoting wound healing in diabetic rats (62). Remarkably, we observed that, compared to the BR gel, the BR–PolyET–NC gel enabled the replacement of the damaged tissues at the wound site and complete reversal of the wound area with normal tissues on the 15th day of treatment.

#### 3.5.2 Bacterial colony counts at wound site

The antibacterial effect of BR–PolyET–NC at the wound site was compared to BR gel, blank gel, and control (untreated) is shown in Figure 6C. The bacterial colony was measured lowest for the BR–PolyET–NC gel, followed by BR gel and blank gel. The colony counts for a group of animals treated with BR–PolyET–NC gel, BR gel, and blank gel and negative control were noted. The highest and lowest numbers of colony counts on the 15th day were found to be for negative control and BR–PolyET–NC, respectively. The bacterial colony counts of the BR–PolyET–NC gel on 5th, 10th, and 15th days were recorded to be 15, 17, and 6 CFU, respectively compared to 26, 35, and 20 CFU for the BR gel. The bacterial count on these days at the wound site in the case of the BR–PolyET–NC gel was significantly lower than the BR gel (*p* < 0.05), indicating the potential of therapeutic use in wound healing therapy (61).

#### 3.5.3 Histopathogical investigation of the wound

**Figure 7:**
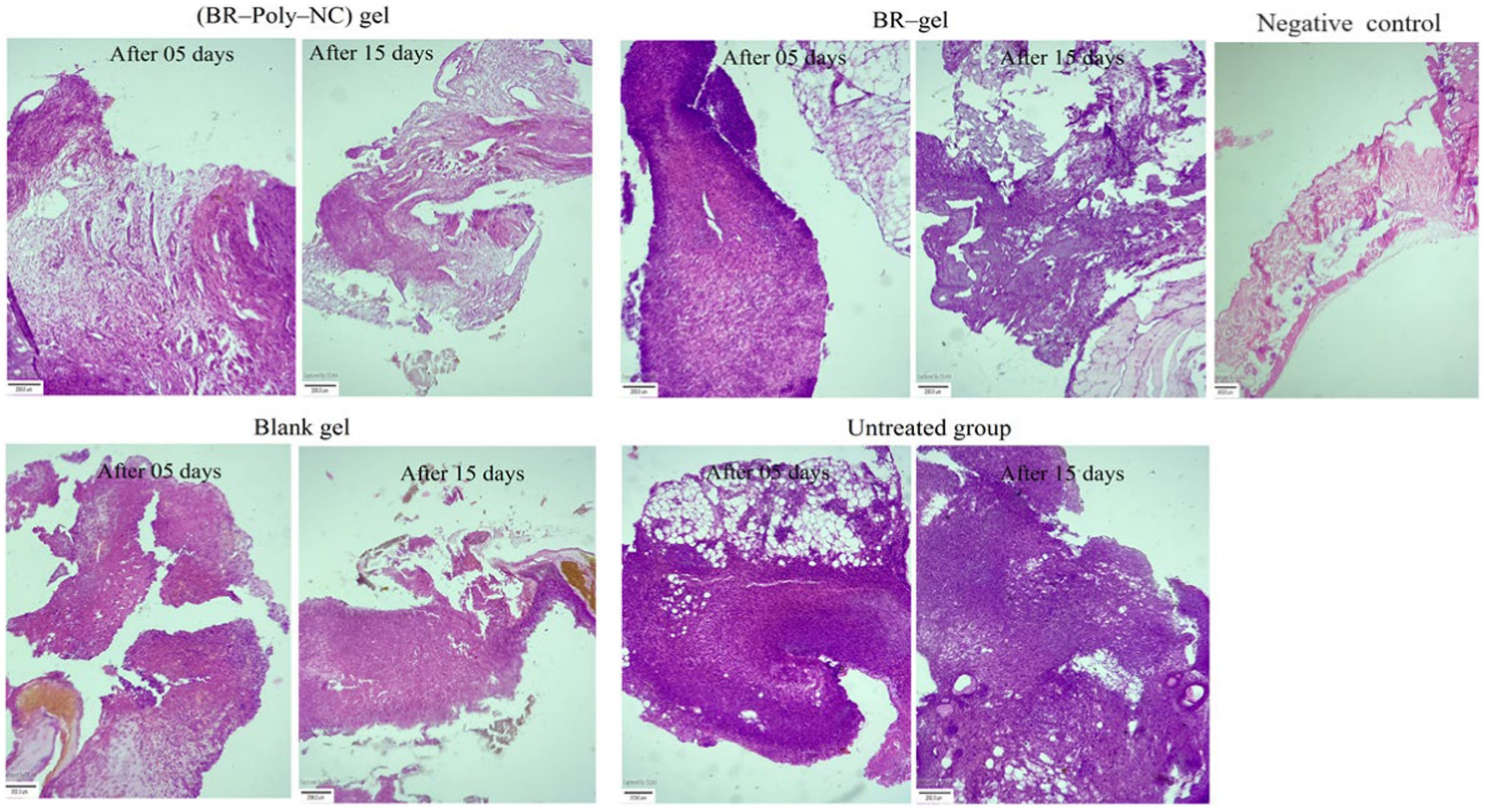
Histopathological examination of various treatment groups showing the changes from the 5th day to the 15th day of therapy in BALB/c mice: BR–PolyET–NC gel treated group; BR gel treated; blank gel treatment group; and untreated or positive control and negative or normal at the end of the 15th day. The BR–PolyET–NC gel treated group stated the great influence in wound healing capability as demarcated by keratinization, formation of hair follicles, or skin appendages. The blank gel and BR gel groups indicated acanthosis and follicular hyperkeratosis and inflammatory infiltrates in dermal tissues. The connective fibrous tissue marks the existence of inflammatory infiltrates. Scale bar: 400 μm.

Histopathology is an important parameter for investigating the wound microenvironment and the healing process. Histolopathogical images of the wound site are expressed in Figure 7. The microscopy assessment of wound tissue calls for the measurement of macrophagic cells, neutrophil cells, fibroblast, fibrocytes, and collagen cells in the animal groups included for wound study. The microscopic evaluation of the histopathology in various treated groups are shown in Figure 6. The observations revealed that the BR–PolyET–NC treated group of animals had the best re-epithelization in the wound healing process and achieved the highest percentage of healing compared to the BR gel treated group in the entire treatment schedule of 15 days in BALB/c mice. The BR–gel treated group of animal showed better result in healing process compared to placebo gel and untreated group. The process of re-epithelization was observed to begin on 5th day and, consequently, wound closure completed on 15th day. The BR–PolyET–NC treated group showed statistically significant difference in the wound closure rate, i.e., >99% of the wound closed on the 15th day, followed by the BR gel and blank gel treated groups and the untreated group.

Microscopic studies demonstrated that the treatment outcome of the BR–PolyET–NC gel treated group was excellent and statistically significantly different compared to other groups, including the BR gel treated group. The haematoxylin and eosin (H and E) stained slides of various treated groups illustrate the re-epithelization and normalization of the wound treated skin. It was further disclosed that the number of neutrophils and macrophages significantly increased on 4th day and then their number significantly decreased on 7th day onwards for the entire group, but the decline was observed to higher in the BR–PolyET–NC treated group. The inflammatory infiltrate greatly reduced in the early phase in the BR–PolyET–NC treated animals compared to the BR gel treated group and other groups. Furthermore, an increased number of macrophages was observed until 7th day, and thereafter, it decreased on 14th day in all the groups, and the decrease was more pronounced in the BR–PolyET–NC treated group than other groups. Moreover, a steep increase in fibroblasts and fibrocytes was observed on 7th day, indicating re-epithelization, and then a sharp decline on 14th day of treatment in the BR–PolyET–NC treated group compared to other groups (63).

The epidermal layer of the control group shows evidence of stern acanthosis and follicular hyperkeratosis, as depicted in Figure 7. On the other hand, the dermal layer bearing an ample number of fibers from the extracellular matrix such as collagen fibers, disperse blood vessels, and large chronic inflammatory infiltrates such as macrophages, lymphocytes, and B and T cells. These characteristics of hispathological alteration skin tissues are a clear indicative of chronic wound. In the BR–PolyET–NC gel treated group, as shown in Figure 7, the epidermal, dermal, and hypodermal tissues showed normal characteristics after treatment compared to the control group.

Acanthosis and follicular hyperkeratosis were absent in the epidermis layer. The dermal tissue bears bundle fibers of collagen, muscle fibers, and skin appendages. Fatty tissues and blood vessels are shown in the hypodermal layer. In the BR gel and blank gel treatment groups (Figure 7), the epidermis layer indicated mild acanthosis as well as follicular hyperkeratosis. The dermal layer showed muscle fibers, bundle fibers of collagen, skin appendages, less inflammatory infiltrates, and lymphocytes. The hypodermis layer depicted subcutaneous fatty tissues and blood vessels (42).

In the early inflammatory phase of the healing process after 5th day, polymorphonuclear cells such as neutrophil infiltration trigger the recruitment of monocytes on the wound site, which later leads to the formation of macrophages. Following this, the proliferative phase begins with the formation of the granulation tissues, fibroblasts, and angiogenesis cells. The appearance of different cells such as lymphocytes, monocytes, angiogenesis, and fibroblast cells at the wound site supports the healing progress with the formation of new blood vessels and capillaries.

The numbers of monocytes and lymphocytes were largely reduced once the healing progression reached a peak level and disappeared completely at the end of the 15th day. The fibroblast cell number significantly reduced after the 10th day and the percentage of re-epithelization increased after the 7th day, leading to the formulation of epidermal cells around wound edges. As shown in the macroscopic structure of the wound covered almost completely on the 15th day. Collagen deposition in the later stage of wound healing is an important stage toward the normalization of cells, and the deposition was more pronounced in the advanced phase of healing. Overall, the results suggested that animals in the BR–PolyET–NC treated groups showed rapid recovery in the healing process involving different transition phases such as inflammatory and proliferative ones, and re-epithelization of, collagen deposition at, neovascularization of, and normalization of the wound site indicates wound closure (64, 65).

Generally, wound healing is a complex physiological process mediated through various cells such asgrowth factors, cytokines, and chemokines (1). These cells may directly or indirectly be involved in the healing process, which is triggered by various imbricating phases, i.e., hemostasis, inflammation, proliferation, and remodeling of tissues to achieve proper healing of the intended site (38). The first response to the injury site starts with hemostasis, which leads to the arresting of bleeding and hemorrhage. Thereafter, an aggregation of platelets and inflammatory cells accumulates at this site and binds with newly formed collagen tissues in the extracellular matrix (ECM). Platelets secrete proteins such as vitronectins, fibronectins, and Sphingosine-1-Phosphate which help in fibrin clots, accelerates vasocontriction, and stops bleeding. They further assist in supplying inflammatory cells, which harbor cell scaffolds that produce chemokines and cytokines that orchestrate the early phase of wound repair (66, 67).

### 3.6 Stability studies

The different assessed parameters of BR–PolyET–NC gel such as particle size, PDI, pH, viscosity, spreadability and extrudability under stability studies of 2 months at elevated and at refrigerated temeperatures are listed in Table 4. After inspecting the gel, changes in pH and viscosity of the gel were not observed; however, less marked changes in particle size and PDI were observed under the stated condition in either of the temeperature. Furthermore, the spreadability was not changed and extrudability was in good conditon. The external feature showed no turbidity or phase separation. The stability experiment unveils that optimized BR–nanocomposite gel was physico-chemically stable for 2 months. This could be due to the complexing agent/gelling agent at elevated temperatures, which raised the free energy of the gel system, resulting in collision and thin particle aggregation. The stability study confirmed that the stable nanocomposite gel can safely preserve for 2 months at this temeperature (68–70).

**Table 4:** Stability appraisal characteristics of the BR–PolyET–NC gel at elevated temeprature (40 ± 2°C/75 ± 5% RH) and at refrigerated temperature (5 ± 3°C).

At elevated temperature	Time (Days)	pH	Paticle size, nm	PDI	Viscosity, Pa.s	Spreadability	Extrudability
	0	6.3 ± 0.01	71 ± 3.5	0.45	9.23	6.20 ± 0.12	Good
	30	6.3 ± 0.03	73 ± 6.5	0.46	9.32	6.25 ± 0.15	Good
	60	6.4 ± 0.02	75 ± 5.2	0.47	9.26	6.23 ± 0.13	Good
At refrigerated temperature	0	6.3 ± 0.01	71 ± 3.5	0.45	9.23	6.20 ± 0.12	Good
	30	6.3 ± 0.01	72 ± 2.3	0.44	9.24	6.23 ± 0.20	Good
	60	6.4 ± 0.03	73 ± 4.2	0.46	9.27	6.24 ± 0.18	Good

## 4 Conclusion

The BR-loaded BR–PolyET–NC nanocomposite was developed for improved BR delivery and long-acting efficacy of the involved drug in the wound healing process. The nanocomposite of BR was prepared by applying the ionic gelation/complexation technique and then loaded into the gel. A robust and stable formulation was obtained using a three-level, three-factor experimental design. The optimum composition of the developed formulation comprising chitosan (X1 = 58.5 mg), sodium alginate (X2 = 27 mg), and calcium chloride (X3 = 45.27 mg) had the mean particle size and %EE of 71 *±* 3.5 nm and 91 *±* 1.6%, respectively. The drug release behavior was controlled over a period of 72 h to maximize the therapeutic effect and fasten wound healing. The electron microscopy of the nanocomposite revealed distinct and de-aggregated particles of uniform shape and size, indicating the stability of the nanosystem. The *in vivo* model proved that the developed formulation act upon by declining inflammation, depositioning collagen fibers, enriching blood supply to the wound, and replacing damaged tissues and cell debris. Overall, our study highlights the important use of berberine in the nano-platform in wound healing application, and we believe that it could be translated clinically.

## Data availability statement

The original contributions presented in the study are included in the article/supplementary material, further inquiries can be directed to the corresponding authors.

## Author contributions

MHA, SR, and LA-K: conceptualization. MHA and MS: methodology. MHA and HK: software. SA, NA, and MSA: validation. MHA, GK, and SR: formal analysis. MHA, HK, and AA: investigation. MR and HK: resources. HK and SR: data curation. MHA and LA-K: writing–original draft preparation. A-HE, GK, and MJ: writing–review and editing. MS and MR: visualization. LA-K and MHA: supervision. MHA: project administration. SR, A-HE, and MJ: funding acquisition. All authors contributed to the article and approved the submitted version.
